# The role of oxidative stress in the development of knee osteoarthritis: A comprehensive research review

**DOI:** 10.3389/fmolb.2022.1001212

**Published:** 2022-09-20

**Authors:** Lin Liu, Pan Luo, Mingyi Yang, Jiachen Wang, Weikun Hou, Peng Xu

**Affiliations:** Department of Joint Surgery, HongHui Hospital, Xi’an Jiaotong University, Xi’an, China

**Keywords:** knee osteoarthritis, oxidative stress, reactive oxygen species, cartilage, synovitis

## Abstract

Knee osteoarthritis (KOA) is one of the most common degenerative diseases, and its core feature is the degeneration and damage of articular cartilage. The cartilage degeneration of KOA is due to the destruction of dynamic balance caused by the activation of chondrocytes by various factors, with oxidative stress playing an important role in the pathogenesis of KOA. The overproduction of reactive oxygen species (ROS) is a result of oxidative stress, which is caused by a redox process that goes awry in the inherent antioxidant defence system of the human body. Superoxide dismutase (SOD) inside and outside chondrocytes plays a key role in regulating ROS in cartilage. Additionally, synovitis is a key factor in the development of KOA. In an inflammatory environment, hypoxia in synovial cells leads to mitochondrial damage, which leads to an increase in ROS levels, which further aggravates synovitis. In addition, oxidative stress significantly accelerates the telomere shortening and ageing of chondrocytes, while ageing promotes the development of KOA, damages the regulation of redox of mitochondria in cartilage, and stimulates ROS production to further aggravate KOA. At present, there are many drugs to regulate the level of ROS, but these drugs still need to be developed and verified in animal models of KOA. We discuss mainly how oxidative stress plays a part in the development of KOA. Although the current research has achieved some results, more research is needed.

## Introduction

Osteoarthritis is the most common joint degenerative disease among adults in the world, affecting approximately 78 million people worldwide by 2040 (Hootman et al., 2016; Sharma, 2021). Due to the increase in stress in the weight-bearing part of the joint, long-term strain will lead to cartilage exfoliation, hyperosteogeny, synovial hyperplasia, degeneration, etc. This series of changes is called joint degenerative disease (Abbasi, 2017). Most of a person’s weight is distributed across their knees, making the knee a very important joint, so knee osteoarthritis (KOA) is also one of the most common degenerative diseases (Michael et al., 2010). At present, there is no effective treatment for KOA, but various methods are used to delay the progression of KOA. When KOA develops to the end stage, total knee arthroplasty is generally used to improve the living conditions of patients (Quinn et al., 2018). However, total knee arthroplasty is expensive, so with the development of the ageing world, the prevention and treatment of KOA have increased the burden on society and patients.

Redox biological reactions have the two characteristics of promoting physiological signal responses as well as promoting pathophysiological cues (Espinosa-Diez et al., 2015; Zhang et al., 2022). Oxidative stress is a state of imbalance that causes more reactive oxygen species (ROS) to be produced or reduces the body’s natural antioxidant defences (Kimball et al., 2021). ROS are a class of substances containing oxygen free radicals, which have unpaired electrons that make them unstable and highly reactive; hydrogen peroxide (H_2_O_2_), hydroxyl (OH^−^) radicals, superoxide (O_2_^−^) anions, and nitric oxide (NO) are all examples of reactive oxygen species (Trachootham et al., 2008). Mechanical and chemical stress can lead to an increase in the production of oxygen free radicals, resulting in oxidative damage to tissue (Cannizzo et al., 2011). The excessive production of ROS leads to damage to macromolecules such as protein, fat and DNA (Dröge, 2002; Cutler, 2005; Espinosa-Diez et al., 2015). When free radical production exceeds cellular scavenging, lipid peroxidation, for instance, can be brought on by an overabundance of hydroxyl radicals and peroxynitrite, which destroys cell membranes and lipoproteins (Pizzino et al., 2017).

Articular cartilage deterioration and destruction is the hallmark of KOA, which affects all tissues of the knee joint (Loeser et al., 2012). Cartilage degeneration in KOA is caused by the destruction of the dynamic balance of chondrocytes caused by activation based on other different aspects, in which the matrix degrades enzyme production and exceeds the ability of chondrocytes to secrete matrix components (Bolduc et al., 2019). Elevated levels of ROS and oxidative stress in chondrocytes play a role in the development of KOA (Blanco et al., 2011). This article reviews which pathological reactions are mainly involved in the pathogenesis of KOA by oxidative stress.

## Pathogenesis of knee osteoarthritis

**FIGURE 1:**
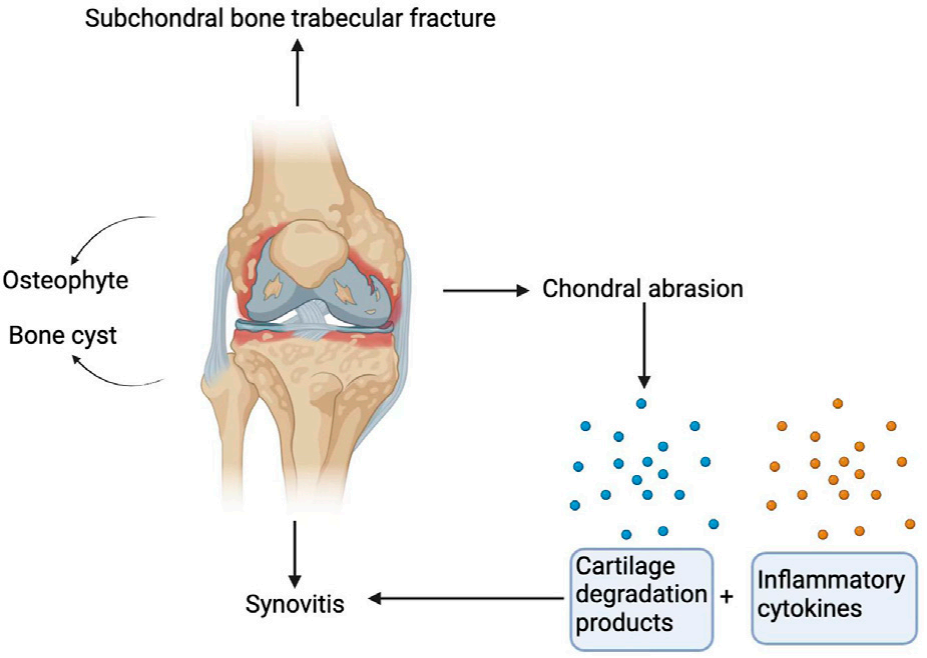
Pathological manifestations of osteoarthritis of the knee joint. Damage to hyaline articular cartilage is the main cause of osteoarthritis. In addition to cartilage lesions, KOA is accompanied by subchondral trabecular fracture, and bone cysts and osteophytes are also characteristics of KOA. Cartilage degradation products are produced after cartilage injury. These cartilage degradation products and other inflammatory factors act on the synovium to release preinflammatory products to induce synovitis.

Among the various structures that make up the knee joint, damage to hyaline articular cartilage is the main cause of osteoarthritis. One of the most obvious risk factors for KOA is ageing, in which ageing of cartilage and chondrocytes plays an important role in the pathogenesis and development of KOA (Rahmati et al., 2017). At first, the surface of the cartilage becomes worn, and with the continuation of the pathological process, deep cracks related to the shedding of cartilage fragments gradually forms, causing delamination and exposure of the calcified cartilage and bone below (Burr and Schaffler, 1997; Burr, 2004). In addition to cartilage lesions, KOA is accompanied by changes in subchondral trabecular structure and bone mass, osteophytes, bone marrow lesions, and the development of cysts (Sandell, 2012) (Figure 1). In addition, the decrease in bone tissue hardness may lead to cartilage deformation and cartilage pathology, which are associated with osteoarthritis (Stewart and Kawcak, 2018).

Additionally, synovitis is crucially involved in the development of KOA. Synovitis is characterized by synovial hyperplasia and diffuse infiltration of T and B lymphocytes (Scanzello and Goldring, 2012). Magnetic resonance imaging and ultrasound imaging studies have confirmed that synovitis is positively correlated with the risk of osteoarthritis progression (Baker et al., 2010). Additionally, cartilage damage and malfunctioning chondrocytes contribute significantly to the onset of synovitis. Chondrocytes release matrix metalloproteinases to degrade the cartilage matrix and release cartilage degradation products, which, together with other proinflammatory cell derivatives, act on preinflammatory products of synovium. These preinflammatory products are fed back to chondrocytes to further affect the regulation of their function (Glyn-Jones et al., 2015) (Figure 1).

## Overview of oxidative stress

**FIGURE 2:**
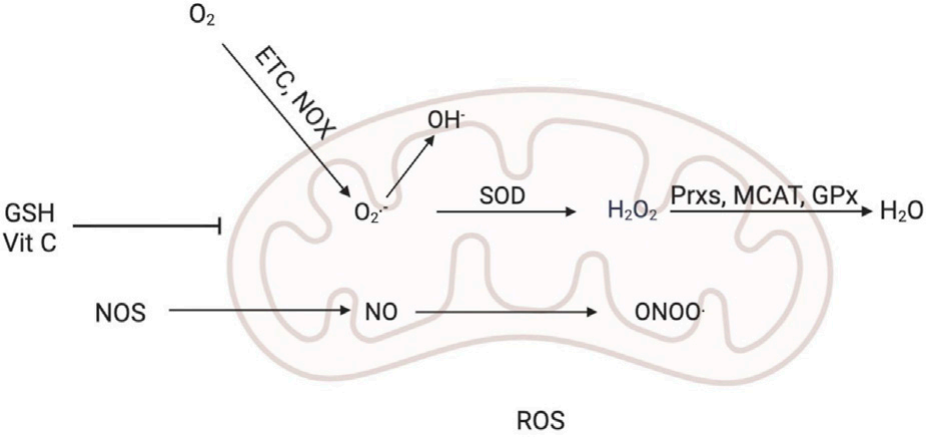
Generation and regulation of ROS. Superoxide (O_2_^−^) is produced by incomplete reduction of molecular oxygen in the mitochondrial electron transport chain (ETC) or through NADPH oxidase (NOX) activity. In general, SOD disproportionates O_2_^−^ to form H_2_O_2_, and peroxidase (Prxs) further reduces H_2_O_2_ to water, catalase (CAT) or glutathione peroxidase (GPx). SOD can also be converted into OH^−^, and OH^−^ is a more reactive and destructive ROS. NO is produced by three different NO synthases (NOSs). O_2_^−^ reacts with NO to produce ONOO^−^, which leads to the formation of nitrotyrosine. Reduced glutathione (GSH) and ascorbic acid (vitamin C) can reduce ROS levels.

O_2_^−^ is the most abundant oxygen free radical under physiological conditions, and mitochondria are thought to be the primary source. O_2_^−^ is a potent reactive oxygen species that has a strong effect on the redox state of cells. Not only is O_2_^−^ crucial for a normal immune response, but its direct oxidation of proteins also has far-reaching effects on signal transduction, gene expression, and the cell cycle (Turrens, 2003). To convert O_2_^−^ to H_2_O_2_, superoxide dismutase (SOD) is needed. O_2_^−^, produced by the mitochondrial electron transport chain or by nicotinamide adenine dinucleotide phosphate (NADPH) oxidase (NO_x_) activity. By converting O_2_^−^ to OH- in the presence of Fenton reactive metals, SOD can also generate a more reactive and destructive ROS, hydroxyl radical (OH^−^). (Dickinson and Chang, 2011). NO is produced by three different NO synthases (NOSs): inducible NOS (iNOS), endothelial NOS (eNOS) and neuronal NOS (nNOS). NO derivatives can cause macromolecular cell damage and nitrosation stress; for example, O_2_^−^ combines with NO to produce ONOO-, which leads to the synthesis of nitrotyrosine, another posttranslational alteration signalling oxidative stress and injury (Oh et al., 2017). One of the illnesses of ageing is osteoarthritis, and nitrotyrosine is found in joints where there is wear and tear (Carlo and Loeser, 2003). Superoxide (O_2_^−^) and hydrogen peroxide (H_2_O_2_) are the most typical ROS types in chondrocytes with ageing and osteoarthritis. Peroxynitrite (ONOO-), an NO product, is also present in cartilage and helps control how chondrocytes work (Bolduc et al., 2019). As ROS production rises, the defence system of the cell against oxidative stress is triggered, leading to efficient clearance of ROS molecules. Multiple enzymes, such as mitochondrial catalase (MCAT), peroxidases (Prxs), glutathione peroxidase (GPx), SOD and nonenzymes, such as glutathione and ascorbic acid (vitamin C) (Pisoschi and Pop, 2015), work together to form the antioxidant defence mechanism of the cell. Glutathione is a small molecular mercaptan that plays a key role in oxidative metabolism (Figure 2). Sufficient levels of glutathione must be maintained to exert protective and biosynthetic functions (Wang et al., 2021). Glutathione is essential for maintaining proper cellular redox potential and protecting against oxidative damage. It is important to note that glutathione can be either reduced (GSH) or oxidized (GSSG). Maintaining redox homeostasis and delivering antioxidant stress protection are dependent on the total glutathione concentration and the GSH/GSSG ratio in cells (Diaz-Vivancos et al., 2015). Prxs receive new oxygen at the mercaptan active site to protect proteins from oxidation by hydrogen peroxide (Rhee et al., 2012). By oxidizing GSH, GPx protects membrane lipids from H_2_O_2_-induced oxidation (Lubos et al., 2011).

## The role of oxidative stress in articular cartilage degeneration

**FIGURE 3:**
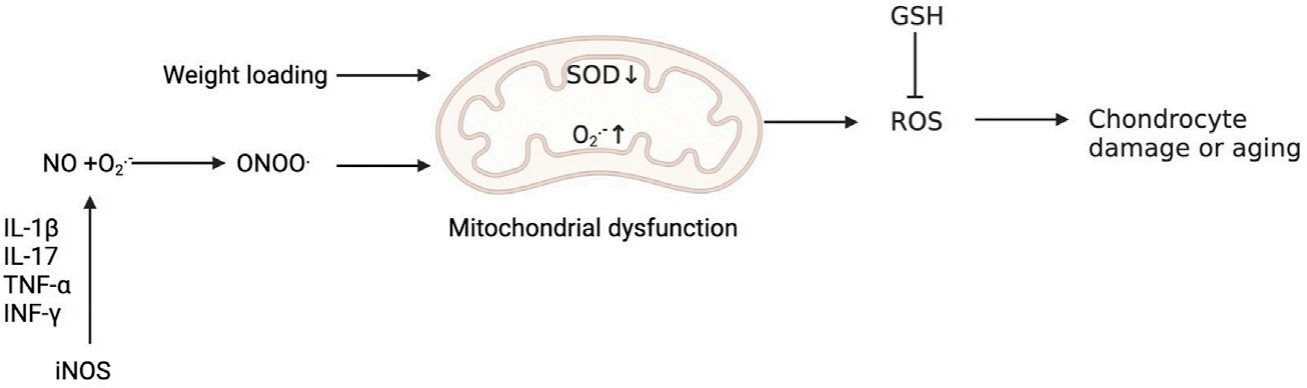
Oxidative stress is involved in the injury and senescence of chondrocytes. Weight loading promotes the production of O_2_^−^ in mitochondria and reduces the expression of SOD, while mitochondrial dysfunction induced by ROS in mitochondria will further lead to cartilage degeneration. The production of NO by activating iNOS is initiated by signals from proinflammatory cytokines, including IL-1β, IL-17, tumour necrosis factor-α (TNF-α) and interferon-γ (INF-γ). O_2_^−^ reacts with NO to produce ONOO^−^. Peroxynitrite can induce mitochondrial dysfunction, resulting in chondrocyte damage. Reduced glutathione (GSH) can reduce the level of ROS.

Biochemical analysis of degenerative cartilage from patients with OA showed that there was a pathological relationship between the downregulation of SOD2 and cartilage degeneration in the progression of OA, suggesting that the redox balance centred on SOD2 in mitochondria plays a central role in the pathogenesis of KOA (Ruiz-Romero et al., 2009; Scott et al., 2010). We all know that increased weight loading is one of the main risk factors for KOA. Koike et al. have shown that mechanical load *in vivo* promotes the production of O_2_^−^ in mitochondria of chondrocytes, and the expression of SOD1 and SOD2 in mitochondria decreases, while mitochondrial dysfunction induced by superoxide in mitochondria will further lead to cartilage degeneration (Koike et al., 2015). In addition, the expression of all three SODs was shown to be high in human cartilage but dramatically reduced in advanced OA cartilage (34), which further aggravated the oxidative stress response in chondrocytes and promoted the degeneration of chondrocytes (Scott et al., 2010). The decrease in SOD2 in OA chondrocytes is related to the increase in promoter methylation (Scott et al., 2010). Extracellular SOD has also been shown to decrease in human OA cartilage, suggesting that extracellular SOD also plays a key role in regulating ROS in cartilage (Regan et al., 2005). (Figure 3) Some studies have shown that Nox is involved in macrophage phagocytosis and neutrophil bactericidal activity, so Nox has a firmly established importance in immune function (Dang et al., 2002; Brandes et al., 2014). In addition, O_2_^−^ and H_2_O_2_ produced by Noxs in many cell types are essential for normal signal transduction of growth factors and cytokines (Holmström and Finkel, 2014). For example, ROS produced by Nox2 and Nox4 is involved in chondrocyte differentiation (Kim K. S. et al., 2010). But other researchers have found Nox4 is considered to be the main active subtype of chondrocytes in OA cartilage, in which the activation of Nox4 by proinflammatory cytokines increases the production of O_2_^−^and H_2_O_2_ (Morel et al., 2015). The formation of ROS caused by the activation of Nox4 aggravates the decomposition of cartilage (Rousset et al., 2015).

NO in cartilage and synovium is produced by iNOS, which mediates the expression of inflammatory factors, inhibits the synthesis of collagen and proteoglycan, and induces chondrocyte apoptosis and pain (Suantawee et al., 2015). The selective inhibition of iNOS reduces the tissue level of catabolic factors, so NO plays an inflammatory role in OA (Ahmad et al., 2020). The production of NO in OA cartilage is unusually high because OA chondrocytes produce high levels of NO. NO regulates ECM homeostasis and cytokine expression, leading to oxidative damage and chondrocyte apoptosis, thus promoting the pathogenesis of OA (Scher et al., 2007). In addition, excessive NO produced by iNOS leads to cartilage injury by enhancing matrix metalloproteinase (MMP) activity and down regulating proteoglycan and collagen biosynthesis (Lepetsos and Papavassiliou, 2016). In addition, by reacting with oxidants such as superoxide anions, NO promotes cell injury and makes chondrocytes vulnerable to apoptosis induced by cytokines (Amin et al., 2000).

One study found that NO alone did not result in chondrocyte death but that NO triggered apoptosis when it reacted with O_2_- to produce chondrocytes (Blanco et al., 1995); therefore, it seems that NO can increase chondrocyte death through apoptosis (Del Carlo and Loeser, 2002). The flexibility of cartilage is due in large part to the presence of proteoglycans in the cartilage matrix. NO may disrupt cartilage homeostasis by inhibiting proteoglycan production (Clancy et al., 1997). The production of NO by activating iNOS is initiated by signals from proinflammatory cytokines, including interleukin (IL)-1β, IL-17, tumour necrosis factor-α and interferon-γ (Nathan and Xie, 1994). Martel-Pelletier et al. found that the expression of iNOS and the increased level of the downstream product NO in chondrocytes work together to maintain the role of inflammatory cytokines, which will further cause chondrocyte damage (Martel-Pelletier et al., 1999). O_2_^−^ reacts with NO to produce ONOO^−^. Peroxynitrite can induce mitochondrial dysfunction through a calcium-dependent process, which leads to chondrocyte apoptosis mediated by calpain (Whiteman et al., 2004).

Zhu et al. discovered that the redox balance and glutathione content were drastically altered depending on the presence and pattern of load-rest cycles. For example, loading without rest for 48 h under physiological cycle conditions caused significant net oxidation of glutathione in cartilage, which decreased the protection of glutathione against further oxidative stress (Zhu et al., 2020). One study found that Prx3 (mitochondrial Prx) is highly oxidized in the cartilage of elderly patients with osteoarthritis, indicating that oxidative stress is intensified in degenerative cartilage. Overproduction of ROS can be caused by mitochondrial malfunction or unchecked SOD2 expression, which in turn can irreparably damage chondrocytes and trigger cell death via apoptosis or necrosis (Lepetsos and Papavassiliou, 2016).

## The role of oxidative stress in synovitis

The aetiology of KOA is heavily influenced by synovitis. Inflamed synovium produces prostaglandins, leukotrienes, ROS, cytokines, chemokines, and adipokines, all of which contribute to cartilage breakdown and further exacerbate inflammation (Scanzello and Goldring, 2012). Increased synovitis and angiogenesis are related to oxidative stress induced by hypoxia (Biniecka et al., 2010). To reduce oxidative stress and hypoxia-induced mitochondrial mutation in inflammatory arthritis, tumour necrosis factor (TNF) blocking treatment is effective (Biniecka et al., 2011b). Low synovial oxygen supply efficiency in an inflammatory environment is caused by a combination of factors, including an imbalanced network of synovial microvessels and the increased energy demand of activated infiltrating immune cells and resident inflammatory cells. This combination of factors, in turn, causes a hypoxic microenvironment and mitochondrial dysfunction (McGarry et al., 2018), promoting inflammation and oxidative damage by increasing the production of ROS (McGarry et al., 2018). During the process of oxidizing nutrients to generate adenosine triphosphate (ATP), mitochondria produce ROS (Kuksal et al., 2017). Changes in mitochondrial DNA (MtDNA) in somatic cells are caused by elevated oxidative stress because the mitochondrial genome is very susceptible to mutation (Biniecka et al., 2011a; Harty et al., 2012). The proinflammatory mitochondrial phenotype is related to mtDNA mutation and decreased oxygen partial pressure in the synovium (Du et al., 2020), suggesting that hypoxia and oxidative stress may play a significant role in causing joint inflammation. The inflammatory response of fibroblast-like synoviocytes in rheumatoid arthritis have been shown to be able to be reduced by reducing oxidative stress in peripheral blood mononuclear cells (Lee et al., 2021). Phagocytes produce large amounts of reactive oxygen species during respiratory outbursts, and T cells usually exist near phagocytes. Activated phagocytes produce H 2O 2 through NOX-2. H_2_O_2_ can oxidize mercaptan on the surface of T cells and enter into the interior of T cells. H_2_O_2_ in T cells can oxidize glutathione (GSH) and interfere with DNA synthesis (Belikov et al., 2015). Compared with traditional T cells, T reg cells have lower levels of intracellular ROS and can be protected from H 2O 2 induced death (Mougiakakos et al., 2009). In addition, T cell homeostasis requires the balance of redox reaction. Changing the level of ROS or antioxidants to disrupt this balance will lead to T cell hyperresponsiveness or hyporeactivity, which may lead to the development of various pathology (Gelderman et al., 2007). For example, increasing the level of mercaptan in T cells can lead to T cell-mediated arthritis in mice after collagen immunization (Gelderman et al., 2007).

Abnormal ROS signalling in OA synovial fibroblasts induced by cytokines, thrombin or stress is related to the increased activity of nuclear factor erythroid 2 p45-related Factor 2 (NFE2L2, also known as NRF2) (Bernard et al., 2017). Moreover, Balogh et al. found that oxidative stress in the synovium can promote glycolysis, which may help to accelerate the mechanism of inflammation (Balogh et al., 2018). In addition, Yao et al. found that magnesium ions (Mg^2+^) promote the synthesis of cartilage matrix mediated by hypoxia inducible factor-1α (HIF-1α) (Yao et al., 2019). However, oxidative stress can inhibit the expression of HIF-1α and magnify inflammation, which may impair the therapeutic effect of Mg^2+^ in OA (Mobasheri et al., 2017). The intraarticular combination of Mg^2+^ and vitamin C can reduce oxidative stress and synovitis in OA (Yao et al., 2021). Synovitis worsens, matrix components are destroyed, and apoptosis occurs as a result of oxidative stress-induced mitochondrial and nuclear DNA damage, lipid peroxidation, changes in cellular signal transduction and transcription, and epigenetic transcription factors (Marchev et al., 2017). Andrographis paniculata inhibits lipid peroxidation and nitrate levels to prevent neutrophils from gathering and passing through the cell membrane and reduces the levels of chemokines and inflammatory factors (Luo et al., 2020) .

## The role of oxidative stress in aging of Knee osteoarthritis

Even while being older does not guarantee you will develop osteoarthritis, the changes that come with getting older lay the groundwork for OA to develop in the first place, such as cell senescence and telomere wear, which are thought to represent key mechanisms by which ageing leads to the development of age-related diseases (López-Otín et al., 2013). Mitochondrial failure is an age-related phenomenon that increases intracellular ROS and causes oxidative stress (Venkataraman et al., 2013). Because mitochondria control the ratios of NADH/NAD+, NADPH/NADP+, and GSH/GSSG, alterations in redox homeostasis have been proposed as a potential cause of ageing (Leeuwenburgh et al., 2011).

**FIGURE 4:**
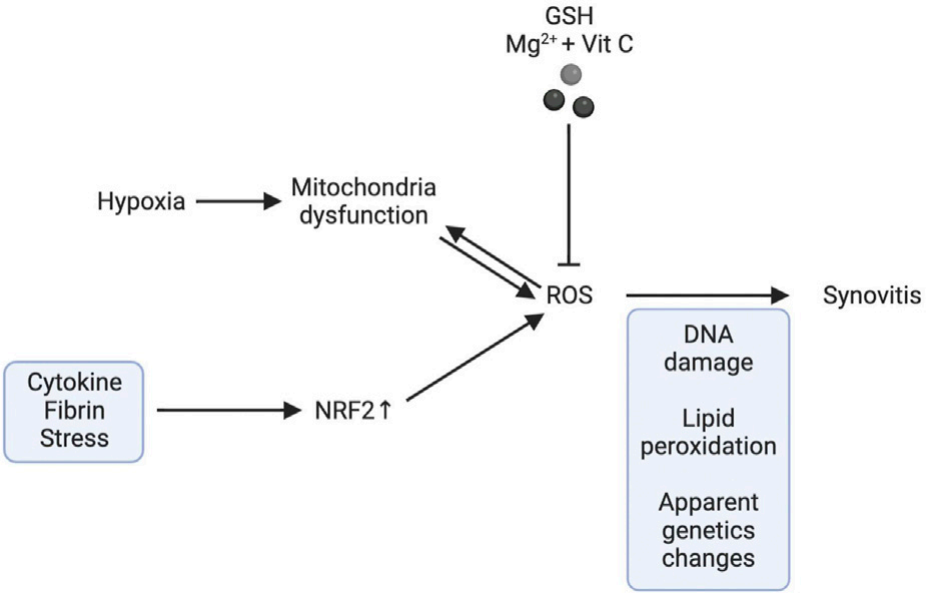
Oxidative stress is involved in the pathogenesis of synovitis. Hypoxia leads to mitochondrial dysfunction, which leads to an increase in the level of ROS and further promotes inflammation. Cytokines, thrombin or stress can cause an increase in nuclear factor erythroid 2 p45-related Factor 2 (NRF2), which leads to an increase in ROS. The combination of Mg^2+^ and vitamin C can reduce oxidative stress and synovitis in OA. At the cellular level, oxidative stress can cause DNA damage, lipid peroxidation and epigenetic changes in gene expression, leading to synovitis deterioration.

**FIGURE 5:**
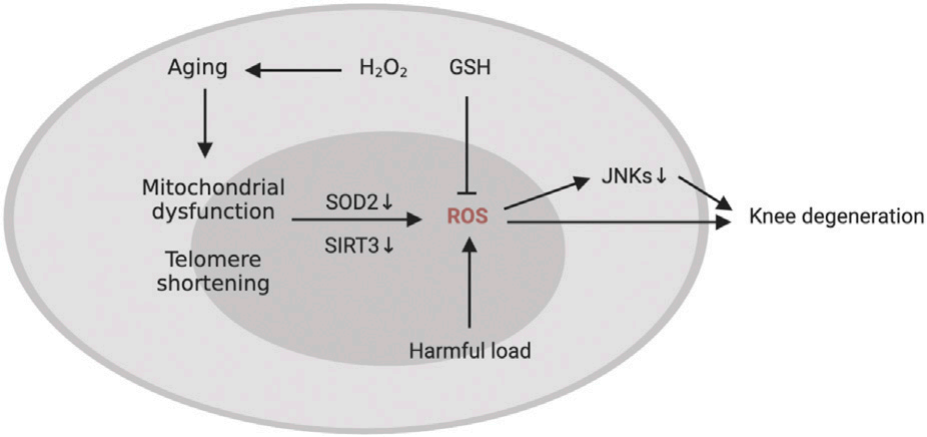
Chondrocyte senescence and oxidative stress. Hydrogen peroxide can cause the ageing of chondrocytes, such as telomere shortening and mitochondrial dysfunction. Sirtuin 3 (SIRT3) protein is lost with ageing, which damages the SOD2 activity of cartilage, affects the regulation of mitochondrial redox in cartilage and stimulates ROS production. In addition, harmful mechanical loads can also stimulate an increase in ROS. Oxidative stress inactivates c-Jun N-terminal kinases (JNKs) and leads to cartilage degeneration. Reduced glutathione (GSH) can combat oxidative stress caused by ageing.

The gradual breakdown of extracellular matrix (ECM) is a hallmark of KOA (Loeser, 2017), which is caused by an imbalance of catabolic and anabolic signals in cartilage. Oxidative stress significantly accelerates the telomere shortening and ageing of chondrocytes (Brandl et al., 2011). In addition, in OA chondrocytes, the decrease in the activity of respiratory chain complexes I, II and III may affect several pathways related to cartilage degradation, including oxidative stress, biosynthesis of chondrocytes, increased inflammation and matrix catabolism induced by cytokines, calcification of cartilage matrix and increased apoptosis of cartilage cells (Blanco et al., 2011). Mutations in mtDNA or the direct impact of proinflammatory mediators such cytokines, prostaglandins, ROS, and nitric oxide may contribute to mitochondrial dysfunction in chondrocytes in KOA (Kim J. et al., 2010) (Figure 4). In addition, proteomic analysis showed that mitochondrial SOD2 in human chondrocytes decreases with age (Ruiz-Romero et al., 2006). Some studies have shown that ageing can lead to an imbalance in the anabolism and catabolism of chondrocytes and promote the senescence and apoptosis of chondrocytes (Loeser et al., 2002; Shane Anderson and Loeser, 2010). Both human and primate ageing cartilage and OA cartilage showed increased generation of hydrogen peroxide and active nitrogen (including NO) (Loeser et al., 2002). Human chondrocyte explants grown in the presence of hydrogen peroxide exhibited senescent features, including telomere shortening, decreased replication ability and decreased glycosaminoglycan production. Fu et al. found that Sirtuin 3 (SIRT3) protein is lost with ageing, which can damage the SOD2 activity of cartilage. Due to the decrease in SIRT3 expression and the impairment of SOD2-specific activity, ageing promotes the development of KOA, impairs the regulation of redox of mitochondria in cartilage and stimulates ROS production (Passos et al., 2010; Fu et al., 2016). Stress-induced chondrocyte senescence and OA may be the result of an increase in ROS generation in cartilage, which may be stimulated by damaging mechanical load (Yamazaki et al., 2003). Collins et al. found that MCAT inhibited the catabolism of chondrocytes induced by toluene diketone and inhibited the progression of age-related osteoarthritis in mouse models (Collins et al., 2016), pointing to the oxidative stress that comes with ageing being able to interfere with the regular physiological signal transduction of the body, which in turn can cause osteoarthritis (Figure 5).

Members of the mitogen-activated protein kinase (MAPK) pathway have been shown in some research to play a role in regulating cell survival and oxidative stress tolerance. For example, oxidative stress inactivates c-Jun N-terminal kinases (JNKs) in human chondrocytes cultured *in vitro* (Nelson et al., 2018). In mice, the loss of JNK1 and JNK2 led to more severe age-related OA and ageing of cartilage and synovium (Loeser et al., 2020), an indication that JNK is a detrimental regulator of joint degeneration. Glutathione, as one of the reductants, is also a regulator of joint senescence in ageing-induced KOA. For example, Carlo et al. found that the ratio of GSH/GSSG in chondrocytes in elderly patients (age ≥50) is lower than the ratio of GSH/GSSG in chondrocytes in young patients (age 18–49), suggesting that oxidative stress increases with age, which increases the risk of oxidant-mediated cell death in chondrocytes through the imbalance of the glutathione antioxidant system (Carlo and Loeser, 2003). Therefore, damage to the glutathione system may lead to proinflammatory-induced oxidative stress in chondrocytes, especially in the process of senescence (McCutchen et al., 2017; Issa et al., 2018). The increase of ROS in chondrocytes caused by aging may be the cause of oxidative damage of genomic and mitochondrial DNA (McCulloch et al., 2017). Mitochondrial DNA damage in turn leads to the senescence of osteoarthritis chondrocytes. The stagnation of chondrocyte proliferation in this case is due to the accumulation of DNA damage after exposure to stress inducers (Minguzzi et al., 2018). Therefore, chronic oxidative stress and mitochondrial dysfunction may be the main causes of chronic degenerative diseases. In addition, researchers have found that the aging of cartilage is not caused by telomere wear and aging of mature chondrocytes, but is attributed to a group of progenitor cells (Fellows et al., 2017). Combining these results, we can see that there is a correlation between oxidative stress and cartilage ageing, which may promote the pathogenesis of KOA.

## How to solve the oxidative stress in Knee osteoarthritis

Several different antioxidant treatment methods are being investigated, some of which are currently in clinical trials. These measures include removing O_2_^−^ before reacting with NO to form ONOO-, removing H_2_O_2_ before forming OH-, using precursors to increase GSH and increasing the synthesis of antioxidant enzymes.

SOD is essential for preventing oxidative stress since it is the sole enzyme that can remove O_2_^−^ from mammalian cells. Since it was discovered in 1969, SOD has generated interest due to its potential as a treatment. Numerous SOD simulations have been created since then. Metalloporphyrins, Mn cyclic polyamines, nitrogen oxides, and other compounds are included in these simulations. Previous research has listed a summary of their chemical characteristics (Batinić-Haberle et al., 2010; Bonetta, 2018). The most comprehensive SOD simulator studied is probably manganese porphyrin. At present, researchers have synthesized various manganese porphyrin compounds and evaluated their O_2_^−^disproportionation activity (Batinic-Haberle et al., 2015). For example, the protective and therapeutic effects of MnTE-2-pYp 5 + and MnTDE-2-ImP5+ have been confirmed in animal models. (Mackensen et al., 2001; Gauter-Fleckenstein et al., 2008; Rabbani et al., 2009; Ganesh et al., 2016). The ability of GC4419 to remove superoxide anions selectively without interacting with other oxidants makes it a further intriguing SOD mimic (Aston et al., 2001), and GC4419 shows therapeutic effects in a mouse model of arthritis (Salvemini et al., 2001). In addition, a variety of GPX simulations have been developed, among which ebselenoline is the best known. Ebselenoline has been shown to reduce oxidative damage in inflammation-related carcinogenesis (Nakamura et al., 2002). However, there are no related experiments to prove that ebselenoline can effectively improve oxidative stress in KOA. One of the most researched medicinal antioxidants is N-acetylcysteine (NAC). There is some indication that NAC supplementation is particularly important in mediating the antioxidant effect of GSH (Rushworth and Megson, 2014). NAC has been used in the treatment of many diseases, including airway cystic fibrosis (Conrad et al., 2015) and nephropathy (Xu et al., 2016). However, exogenous GSH degrades quickly in plasma, and GSH cannot be delivered efficiently to most cells (Wendel and Cikryt, 1980). Therefore, the ester derivative of GSH is a more successful complementary strategy. Many studies have confirmed that GSH esters can efficiently increase GSH in cells and/or tissues in cells and animal models (Chen et al., 2000; Anderson et al., 2004).

Antioxidant enzyme induction of polyphenols is mediated by the NRF2 signal (Forman et al., 2014). Therefore, as a result, NRF2 activator is viewed as a promising medication for boosting antioxidant defences and reducing pathology. Some of the antioxidant enzymes utilized in clinical studies for treating and preventing disease are induced by extracts from foods such as tea, cocoa, and various vegetables and fruits (Pandurangan et al., 2015; Li et al., 2016). For example, for a variety of diseases, including chronic obstructive pulmonary disease, osteoarthritis, joint stiffness and diabetic nephropathy (Yagishita et al., 2019). NOXs, as the source of O_2_^−^ and H_2_O_2_, play an important role in redox signal transduction; however, a problem arises when NOXs are activated to an unhealthy degree and cause harm to healthy tissue. Inhibiting NOX1, NOX2, and NOX4 has been shown to be beneficial in animal models (Teixeira et al., 2017). By contributing an electron to neutralize free radicals, vitamin C is another key antioxidant that plays a role in lowering oxidative stress (Frei et al., 1989). The antioxidant properties of Vitamin E have been demonstrated by numerous other investigations (Hill et al., 2003; Bruno et al., 2006), especially in cases of oxidative stress or other antioxidant deficiencies (Traber and Atkinson, 2007). In animal models, the use of iNOS inhibitors significantly reduced cartilage degeneration and osteophyte formation (Pelletier et al., 1998).

Yamada et al. found that S. tuberculata can reduce the damage related to oxidative stress in serum and reduce the oxidative stress injury and pain caused by knee osteoarthritis in rats (Yamada et al., 2020). In addition, they found that S. tuberculata reduced the damage caused by oxidative stress and cytokines in follow-up studies (Yamada et al., 2022). And the combination of S. tuberculata and photobiologic therapy reduced the levels of cytokines and nitrite/nitrate (Yamada et al., 2022). Pan et al. found that Receptor-interacting protein 2 (RIP2) can regulate cartilage degradation and oxidative stress in IL-1 β-treated chondrocytes by regulating TRAF3 expression and p38-MAPK pathway activation (Pan et al., 2021). TERT-butylhydroquinone can effectively prevent oxidative stress and inhibit apoptosis of rat chondrocytes by activating Nrf2 pathway (Yang et al., 2021). Li et al. found that montelukast can effectively reduce oxidative stress and apoptosis in chondrocytes and improve the viability of chondrocytes (Li et al., 2021). Transforming growth factor β 1 can protect chondrocytes from oxidative stress by regulating autophagy (Kurakazu et al., 2021). Karim et al. found that iron overload in chondrocytes can induce oxidative stress, cell cycle arrest and apoptosis (Karim et al., 2022). Pang et al. found that Bardoxolonemethyl can inhibit chondrocyte apoptosis and ECM degradation induced by oxidative stress *in vitro*, and reduce OA *in vivo* (Pang et al., 2021).

Combined with the above research results, we can use these substances regulating oxidative stress as potential therapeutic drugs for KOA and verify the ability of these substances to regulate ROS in the pathogenesis of KOA in animal experiments.

## Conclusion

Many studies have found that oxidative stress not only promotes the ageing and injury of chondrocytes but also drives the development of synovitis in the pathogenesis of KOA. In addition, with increasing age, the ROS level of chondrocytes increases, which further promotes cartilage injury. An increasing number of studies have found new means to regulate the level of ROS, which provides a new strategy for the prevention and treatment of KOA. Of course, more experiments are needed to study the effect of oxidative stress on KOA.
